# Chemotherapy or Targeted Therapy as Second-Line Treatment of Advanced Gastric Cancer. A Systematic Review and Meta-Analysis of Published Studies

**DOI:** 10.1371/journal.pone.0108940

**Published:** 2014-09-30

**Authors:** Roberto Iacovelli, Filippo Pietrantonio, Alessio Farcomeni, Claudia Maggi, Antonella Palazzo, Francesca Ricchini, Filippo de Braud, Maria Di Bartolomeo

**Affiliations:** 1 Department of Medical Oncology, Fondazione IRCCS Istituto Nazionale dei Tumori, Milan, Italy; 2 PhD Program, Department of Radiology, Oncology and Human Pathology, Sapienza University of Rome, Rome, Italy; 3 Department of Public Health and Infectious Diseases, Statistics Section, Sapienza University of Rome, Rome, Italy; Duke Cancer Institute, United States of America

## Abstract

Chemotherapy is a cornerstone in treatments of gastric cancer, but despite its benefit, less than 60% of patients receive salvage therapy in clinical practice. We performed a systematic review and meta-analysis based on trial data on the role of second-line treatment of advanced gastric cancer. MEDLINE/PubMed and Cochrane Library were searched for randomized phase III trials that compared active therapy to best supportive care in advanced gastric cancer. Data extraction was conducted according to the PRISMA statement. Summary HR for OS was calculated using a hierarchical Bayesian model and subgroup analysis was performed based on baseline Eastern Cooperative Oncology Group Performance Status (ECOG) performance status (0 vs. 1 or more). A total of 1,407 patients were evaluable for efficacy, 908 were treated in the experimental arms, with chemotherapy (231 pts) or with targeted therapies (677 pts). The risk of death was decreased by 18% (HR = 0.82; 95% CI, 0.79–0.85; posterior probability HR≥1: <0.00001) with active therapies. Chemotherapy and ramucirumab were able to decrease this risk by 27% and 22%, respectively. No differences were found between chemotherapy and ramucirumab. In patients with ECOG = 0 a greater benefit was found for chemotherapy with a reduction of the risk of death by 43% and no benefits were found for ramucirumab or everolimus. In patients with ECOG = 1 or more a significant reduction of the risk of death by 32% was reported in patients treated with ramucirumab, even if no significant difference was reported between chemotherapy and ramucirumab. This analysis reports that active and available therapies are able to prolong survival in patients with advanced gastric cancer with a different outcome based on initial patient’s performance status. New trials based on a better patient stratification are awaited.

## Introduction

Gastric cancer (GC) is the third leading cause of cancer death in both sexes worldwide (8.8% of the total), with the highest estimated mortality rates in Eastern Asia and the lowest in Northern America. High mortality rates are also present in both sexes in Central and Eastern Europe, and in Central and South America [1].

Systemic chemotherapy is a cornerstone in treatments of GC both in locally-advanced and metastatic disease. Although no standard regimen for the first-line chemotherapy have been set up on a global scale, its use is associated with a consistent reduction of the risk of death over best supportive care and the combination chemotherapy with cisplatin and fluoropyrimidine (5-FU) improves survival compared to single-agent 5-FU [2].

Considering the new treatment in first-line therapy options, whereas trastuzumab with standard fluoropyrimidine/cisplatin regimen is recommended in patients with HER-2 positive tumors, two and three-drug regimens including 5-FU, cisplatin, with or without an antracyclines, as well as irinotecan or docetaxel-containing regimens are reasonable treatment options for HER-2 negative patients [3], [4].

Despite the majority of patients receive first-line therapy, the analysis of patients enrolled in upfront clinical trials revealed that the attitude towards second-line chemotherapy differ between European and Japanese studies, with a percentage of 14% in REAL-2 and 75% in the SPIRITS study [5], [6].

Regarding patients treated in clinical practice, these percentages are even lower - with only about 45% receiving a salvage treatment compared to Japanese clinical studies. Despite the low number of patients treated in the second-line setting and intrinsic biases, the outcomes of patients receiving a salvage treatment seemed to be influenced positively, with survival times exceeding one year [7], [8].

In recent years, several studies reported that second-line chemotherapy is not the only effective strategy able to increase survival in patients with advanced GC focusing on vascular endothelial growth factor receptor (VEGFR) as new target. In particular, VEGFR-2 is over-expressed in GC tissue as compared to normal mucosa and in presence of lymph nodal metastases [9]. Recent trials reported such as a monoclonal antibody – ramucirumab – or a tyrosine kinase inhibitor – apatinib – against the VEGFR-2 are able to increase the progression free survival and the overall survival in patients treated with one or two previous line of therapies [10], [11].

The aim of this meta-analysis was to estimate the effect of second-line treatment of GC and to analyze the differential role of chemotherapy or targeted agents. We also investigated if different strategies have the same role in patients with different performance status, with the intent to find the best strategy for second-line treatment of this tumor.

## Methods

### Definition of the outcome

For each trial, chemotherapy or targeted therapy as single agents were considered as the experimental treatment and the placebo or the best supportive care (BSC) as the control one. Results were reported for the entire cohort and by type of treatments (chemotherapy, everolimus, ramucirumab), separately.

### Selection of the studies

We reviewed MEDLINE/PubMed and Cochrane Library for citations from January 2004 to February 2014. The search criteria were limited to articles published in English language and phase III clinical trials using appropriate filters available on PubMed. The entry term for the search was “gastric tumor”. During the selection process, search was further restricted to randomized controlled trials in which chemotherapy agents or targeted agent were used as second-line of therapy after first-line platinum- and fluoropyrimidine-based combination therapy over placebo or best supportive care for treatment of advanced gastric cancer. If more than one publication was found for the same trial, the most recent was considered for analysis.

Study quality was assessed by using the Jadad seven-item scale that included randomization, double blinding and withdrawals; the final score was reported between 0 and 5 [12].

### Data extraction

Data’s extraction was conducted independently by two co-authors (R.I. and C.M.) according to the Preferred Reporting Items for Systematic review and Meta-Analysis (PRISMA) statement (**Checklist S1**) [13]; any discrepancies were resolved by consensus between the two authors. The data obtained for each trial were reported in the presented tables, these were: first author’s name, year of publication, trial phase, the number of patients evaluable, the number of arms, drugs used in the experimental and in the control arm, dosage, rate of patients with Eastern Cooperative Oncology Group Performance Status (ECOG-PS) of 0, 1 or 2; and median overall survival (OS) with the relative hazard ratio and 95% CI.

### Statistical method

The HR for OS with the relative 95% CIs was extracted from each study. Summary HR for OS was calculated using a hierarchical Bayesian model [14], where logarithm of study HR was assumed to be normally distributed [15], each study effect was assumed to arise from a Gaussian centered on a study-specific log-HR and the extracted standard error, inflated by 25% to obtain a conservative statement. The study-specific log-HR was assumed to be Gaussian, centered on a pooled log-HR, which is the main object of interest. An informative prior is used for the variance of the pooled HR, as an inverse Gamma centered on an estimator obtained with a moment-based approach (inflated by 25% to obtain a conservative statement). Indirect comparisons were conducted by means of a similar model, assuming an additive shift for the difference of effects on the log-HR scale.

A two-tailed p-value of less than 0.05 was considered statistically significant. All data were collected using Microsoft Office Excel 2007; statistical analyses were performed using R software [16].

## Results

**Figure 1:**
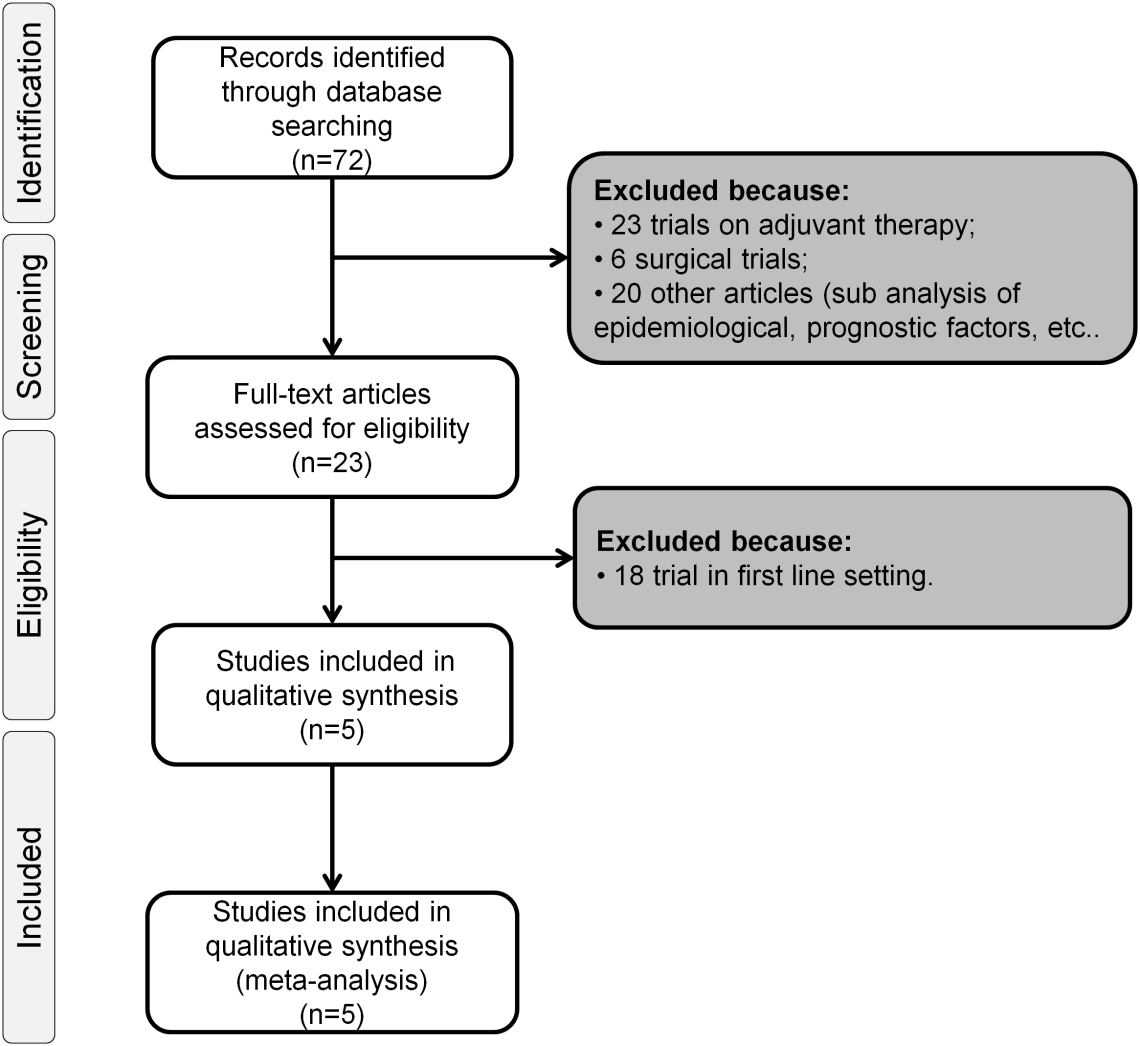
Flowchart of search process.

The electronic search revealed 72 citations. After screening, 49 articles were eliminated because 23 were studies on adjuvant or neoadjuvant therapy, 6 were surgical studies and 20 were related to other aspects of gastric cancer. Among the remaining 23 studies on patients with advanced disease, 18 were eliminated because treatments were administered as first-line. At the end of the review process, only five articles were included in the meta-analysis because of their adequate quality and availability of data (**Figure 1**) [11], [17]–[20]. Among these, only four were considered positive because reached the primary end-point [11], [18]–[20]. The characteristics of each study are presented in **Table 1**.

**Table 1:** Characteristic of the studies included in the analysis.

Author	Year	Experimental arm					Control arm						OS			Jadad’Score
		N°Pts	Therapy	ECOG0	ECOG1	ECOG≥2	N°Pts	Therapy	ECOG0	ECOG1	ECOG≥2		ExpArm	Ctrarm	HR(95% CI)	
Ford HER et al.	2014	84	Docetaxel	28%	55%	17%	84	BSC	26%	60%	14%		5.2	3.6	0.67(0.49–0.92)	2
Fuchs CS et al.	2014	238	Ramucirumab	28%	72%	-	117	Pbo	31%	85%	1%		5.2	3.8	0.78(0.60–1.00)	5
Ohtsu A et al.	2013	439	Everolimus	33%	61%	6%	217	Pbo	32%	55%	12%		5.4	4.3	0.90(0.75–1.08)	5
Kang JH et al.	2012	133	Chemotherapy	54%	46%	-	69	BSC	52%	48%	-		5.3	3.8	0.66(0.48–0.89)	2
Thuss-P. PC et al.	2011	21	Irinotecan	81%		19%	19	BSC	74%			26%	4.0	2.4	0.48(0.25–0.92)	2

### Overall population

A total of 1,424 patients were available for this trial-based meta-analysis and among these 1,407 were evaluable for efficacy. The majority of patients had an ECOG performance status of 0 or 1, being the 95% and 83% in the experimental and control arm, respectively. A total of 908 patients were treated in the experimental arm, and among these 231 received chemotherapy and 677 received targeted therapies. In the experimental arm, the type of chemotherapy used was docetaxel (150 patients) or irinotecan (81 patients), while the targeted therapies were the anti-VEGFR-2 therapy ramucirumab (238 patients) or the mammalian target of rapamycin inhibitor (mTORi) everolimus (439 patients). In the control arm, patients were all treated with BSC with or without placebo.

In the overall population, the active treatment decreases the risk of death by 18% (HR = 0.82; 95% CI 0.79–0.85; posterior probability of HR≥1: <0.00001) (**Table 2**). When analysis was limited to studies reaching the primary end point the reduction of the risk of death was by 27% (HR = 0.73; 95% CI 0.61–0.86; posterior probability of HR≥1: p<0.00001), as reported in **Table 2**.

**Table 2:** Overall Survival in overall population and based on type of studies.

Type of study	Trial	Year	N° of Patients		HR (95% CI)
			Experim. Arm	Control Arm	
Positive studies	Thuss-P. PC et al.	2011	21	19	0.48 (0.25–0.92)
	Kang JH et al.	2012	133	69	0.66 (0.48–0.89)
	Fuchs CS et al.	2014	238	117	0.78 (0.60–1.00)
	Ford HER et al.	2014	84	84	0.67 (0.49–0.92)
	***Subtotal***		***476***	***289***	***0.73 (0.61–0.86)***
Negative studies	Ohtsu A et al.	2013	439	217	0.90 (0.75–1.08)
**TOTAL**			**915**	**506**	**0.82 (0.79–0.85)**

### Survival by type of therapy

When populations were divided based on type of therapy (chemotherapy vs. anti-VEGFR vs. mTORi), chemotherapy was able to decrease the risk of death by 27% (HR = 0.73; 95% CI, 0.58–0.96; posterior probability of HR≥1: 0.00942). On the other hand, treatment with ramucirumab was able to decrease the risk of death by 22% (HR = 0.78; 95% CI, 0.60–1.00), while no significant effect on OS was seen with everolimus (HR = 0.90; 95% CI, 0.75–1.08).

### Survival by performance status

When populations were divided based on ECOG-PS, the 461 patients with ECOG 0 had a greater benefit when treated with chemotherapy over BSC, with a reduction of the risk of death by 43% (HR = 0.57; 95% CI, 0.36–0.91; posterior probability of HR≥1: 0.0092). In this group of patients, no benefit was found for ramucirumab or everolimus over BSC; indirect comparison found a better outcome for patients treated with chemotherapy compared to ramucirumab (posterior probability of chemotherapy worse than or equivalent to ramucirumab: 0.00439) (**Table 3**).

**Table 3:** Overall Survival by type of therapy in patients with ECOG performance status of 0.

Type of therapy	Trial	Year	N° of Patients		HR (95% CI)
			Experim. Arm	Control Arm	
Chemotherapy	Kang JH et al.	2012	72	36	0.59 (0.38–0.90)
	Ford HER et al.	2014	22	19	0.48 (0.24–0.95)
	***Subtotal***		***94***	***55***	***0.57 (0.36–0.91)***
mTOR inhibitor	Ohtsu A et al.	2013	144	70	1.14 (0.81–1.61)
VEGFR inhibitor	Fuchs CS et al.	2014	67	31	1.07 (0.64–1.81)
**TOTAL**			**305**	**156**	**0.88 (0.61–1.28)**

In the 912 patients with ECOG-PS = 1 or more, a trend for greater efficacy was confirmed for those treated with chemotherapy compared to patients treated with BSC, but difference was not strong (HR = 0.80; 95% CI, 0.34–1.89; posterior probability of HR≥1: 0.07). In the same group of patients, a significant effect was found for those treated with ramucirumab over BSC with a reduction of the risk of death by 32% (HR = 0.68; 95% CI, 0.51–0.92; p = 0.04) (**Table 4**). Indirect comparison did not report differences between patients treated with chemotherapy or ramucirumab (posterior probability of chemotherapy worse than or equivalent to ramucirumab: 0.7622).

**Table 4:** Overall Survival by type of therapy in patients with ECOG performance status of 1 or more.

Type of therapy	Trial	Year	N° of Patients		HR (95% CI)
			Experim. Arm	Control Arm	
Chemotherapy	Kang JH et al.	2012	61	33	0.72 (0.46–1.13)
	Ford HER et al. PS = 1	2014	45	50	0.80 (0.53–1.21)
	Ford HER et al. PS = 2	2014	13	12	0.81 (0.36–1.82)
	*Subtotal*		***119***	***95***	***0.80 (0.34–1.89)***
mTOR inhibitor	Ohtsu A et al. PS = 1	2013	269	120	0.86 (0.58–1.08)
	Ohtsu A et al. PS = 2	2013	25	27	1.43 (0.82–2.48)
	*Subtotal*		***294***	***147***	***0.92 (0.70–1.23)***
VEGFR inhibitor	Fuchs CS et al.	2014	171	86	0.68 (0.51–0.91)
**TOTAL**			**584**	**328**	**0.79 (0.64–0.98)**

In summary, regardless of treatment, very little evidence was found for efficacy in patients with ECOG-PS = 0 (HR = 0.88; 95% CI, 0.61–1.28; posterior probability of HR≥1: 0.174), due to the fact that ramucirumab and everolimus do not report a significant decrease of the risk of death, while a benefit was found for chemotherapy. On the other hand, a mild evidence of efficacy was found for patients with PS = 1 or more, with a reduction of 21% (HR = 0.79; 95% CI, 0.64–0.98; posterior probability of HR≥1: 0.015). Indirect comparison indicated that any active therapy over BSC was more effective on patients with ECOG-PS = 1 or more vs. ECOG-PS = 0 (posterior probability of HR in ECOG = 0 better than HR in ECOG = 1: <0.0001), suggesting that patients with symptomatic disease should not be immediately excluded by further lines of therapy.

### Quality of the studies

Jadad’ scores for each trial are listed in Table 1; the mean score was 3.2, confirming the good-quality of the included trials.

## Discussion

First-line treatment of advanced GC with modern regimens confers a benefit of OS exceeding ten months. Even if the addition of trastuzumab to cisplatin-based chemotherapy significantly improved OS in HER-2 positive GC [21], the outcome of the majority of patients is still poor and disease progression invariably occurs.

In metastatic phase, the role of second-line was largely debated because the risk to expose patients to treatment toxicity is high due to performance status deterioration and disease-related symptoms.

Up to date, this analysis is the largest to report that second-line treatment is able to decrease the risk of death by 18%, with a more evident effect in favor of chemotherapy reaching a risk reduction of 27%. Despite the higher absolute benefit of chemotherapy, we are unable to find a relative superiority of this strategy over the targeted agent ramucirumab.

Studies included in this analysis reported that second-line chemotherapy increased the median OS of about two months as compared to BSC. In this context, the choice of the best drugs to use according to efficacy, toxicity and individual patients characteristics remain a open issue. A recent meta-analysis showed that different drugs such as docetaxel or irinotecan, or different administration schedules did not have any influence on outcomes [22]. In facts, the objective response rate and the disease control rate were similar and the decreased risks of death were 29% for docetaxel (HR = 0.71; 95% CI, 0.56–0.90) and 45% for irinotecan (HR = 0.55; 95% CI, 0.40–0.77) over BSC, respectively [22]. Despite this difference, a prospective phase III study comparing weekly paclitaxel to irinotecan as second-line of therapy in GC patients did not show significant differences. The results were a median OS of 8.4 and 9.5 months (HR 1.132; 95% CI, 0.86–1.49; p = 0.38), a median PFS of 2.3 and 3.6 months (HR 1.14; 95% CI, 0.88–1.49; p = 0.33), and an ORR of 13.6% and 20.9% (p = 0.20) for irinotecan and paclitaxel, respectively [23]. Another phase II study that compared the a novel liposomal formulation of irinotecan (PEP02) to standard irinotecan or docetaxel confirmed the lack of any significant advantage in favor of any therapy when compared to other ones [24].

If the role of chemotherapy in second-line treatment of GC was clearly demonstrated, the role of combination chemotherapy over single agent remain an open issue. At least two studies tested this hypothesis comparing two different regimens. The first one compared the activity of irinotecan and 5-FU (FOLFIRI regimen) over irinotecan alone, reporting no significant difference in term of response rate (20.0 vs. 17.2%; p = 0.525), median PFS (3.0 vs. 2.2 months; p = 0.481) or OS (6.7 vs. 5.8 months; p = 0.514) [25]. The second one comparing docetaxel +5′DFUR to docetaxel alone, reported a significant survival benefit for the combination (7.6 vs. 4.0; p<0.05) [26], increasing the confusion in this area. Probably, new large phase III studies will be indispensable in order to better understand the role of combination chemotherapy in the salvage setting.

Until now, two evidence-based strategies are available for patients after first-line of therapy: single agent chemotherapy or targeted therapy such as ramucirumab. Even if the decreased risk of death seems to be higher with the use of chemotherapy in patients with ECOG-PS = 0 and for ramucirumab in patients with ECOG-PS = 1 or more, considering the nature of this analysis, no definitive conclusion may lead in favor of one strategy or the other, while a prospective trial may better address to this fascinating question.

It is noteworthy to mention the results of a recent placebo-controlled, randomized phase III trial that compared weekly paclitaxel and ramucirumab over weekly paclitaxel alone (RAINBOW study). For the first time, in the second-line setting, the addition of a targeted agent to standard chemotherapy demonstrated a significant survival advantage increasing the median OS from 7.36 to 9.63 months (p = 0.0169) and the median PFS from 2.9 to 4.4 months [27]. This positive outcome was reported both in PS = 0 and PS = 1 patients even if a statistical significant benefit was reached only in the PS = 1 patients [27].

Both studies confirm such as the anti-VEGFR2 monoclonal antibody seems to be more active compared to anti-VEGF ones or to anti-VEGFR tyrosine kinase inhibitors. Unfortunately, the reason for these differences is a current challenge and further studies may elucidate the pharmacological differences and probably to improve clinical outcome [28].

Considering the different toxicity profile – of any grade – we account such as ramucirumab was mainly characterized by: fatigue (36%), abdominal pain (29%), decrease of appetite (24%) and vomiting (20%) and by hematological toxicities such as arterial hypertension (16%) and bleeding or hemorrhage (13%) [11]. On the other hand, docetaxel was characterized by the hematological ones such as anemia (28%), neutropenia (16%) other than by specific gastrointestinal (86%), dermatological (42%), or neurological (30%) toxicities [18], [20]. Moreover, the choice of irinotecan seems not improve the toxicity profile of taxanes-based chemotherapy as reported by comparative studies [19], [23], [24].

Even if no definitive data are available about the best treatment strategy for these patients, it is evident that patients’ clinical conditions and co-morbidities, as well as the residual toxicities and the magnitude of benefits from first-line treatment, may all have a role in the choice of second-line therapy.

In this study, we analyzed the activity of different type of therapy in relationship with patient’ performance status at the start of treatment and we found out that the benefit of chemotherapy was more evident in asymptomatic patients with ECOG = 0, with a reduction of the risk of death by 43% (HR = 0.57) as compared to patients with symptomatic disease (HR = 0.80). In this population with suboptimal performance status, the benefit of any type of therapy in terms of decreased risk of death is only by 21%, although it may be increased to 32% with the use of ramucirumab. Probably, a good baseline clinical condition increases the tolerability to chemotherapy and to its related toxicity, on the other hand, the use of a less-toxic approach might be preferred in patients with worse clinical conditions in order to improve quality of life.

This analysis may be influenced by several factor such as the low number of patients – less than 35% – with ECOG = 0 compared to patients with ECOG = 1 or more in the included studies.

Nevertheless, some other limitations may affect these results. First, and foremost, this is a trial-level meta-analysis based on studies and not on individual patient data. Confounding variables such as patient co-morbidities, extent of disease and differences in other possible prognostic factors could not be incorporated into such an analysis. Second, all the included studies were conducted in selected patients with adequate organ function and no severe co-morbidities at the time of study entry. Third, the data on the correlation between ECOG PS and outcomes derived from subgroup analyses of published studies.

## Conclusions

Finally, this study confirms a significant benefit in terms of OS when active second-line treatments are administered to patients with advanced gastric cancer after failure of a previous line of therapy even in patients with impaired performance status.

If the lack of difference between chemotherapy agents was reported by other studies, we suggest a lack of difference between chemotherapy and ramucirumab. Further studies are urgently required to better understand the clinical or molecular characteristic for patient’ selection.

## Supporting Information

Checklist S1 Preferred Reporting Items for Systematic review and Meta-Analysis (PRISMA) statement.
(DOC)
